# Bis(*N*-{bis­[meth­yl(phen­yl)amino]phos­phor­yl}-2,2,2-trichloro­acetamide)di­nitrato­dioxidouranium(VI)

**DOI:** 10.1107/S1600536810005611

**Published:** 2010-02-17

**Authors:** Kateryna O. Znovjyak, Vladimir A. Ovchynnikov, Tetyana Yu. Sliva, Svitlana V. Shishkina, Vladimir M. Amirkhanov

**Affiliations:** a Kyiv National Taras Shevchenko University, Department of Chemistry, Volodymyrska Street 64, 01601 Kyiv, Ukraine; b STC Institute for Single Crystals, National Academy of Science of Ukraine, Lenina Avenue 60, 61001 Khar’kov, Ukraine

## Abstract

In the title compound, [UO_2_ *L*_2_(NO_3_)_2_] {*L* = *N*-{bis­[meth­yl(phen­yl)amino]phosphor­yl}-2,2,2-trichloro­acetamide, C_16_H_17_Cl_3_N_3_O_2_P}, the U^VI^ ions are eight-coordinated by axial oxido ligands and six equatorial O atoms from the phosphoryl and nitrate groups in a distorted hexa­gonal–bipyramidal geometry. There are disordered fragments in the two coordinating *L* ligands: the trichloro­methyl group is rotationally disordered between two orientations [occupancy ratio 0.567 (15):0.433 (15)] in one ligand, and a meth­yl(phen­yl)amine fragment is disordered over two conformations [occupancy ratio 0.60 (4):0.40 (4)] in the other ligand. In the crystal structure, intra­molecular N—H⋯O hydrogen bonds between the amine and nitrate groups are observed.

## Related literature

For the synthesis and structural investigation of the ligand *L*, see: Znovjyak *et al.* (2009 ▶). For a uranium(IV)-containing complex with a similar ligand, see: Amirkhanov *et al.* (1997 ▶). For inter­pretation of the coordination polyhedra of uranium ions, see: Keppert (1982 ▶).
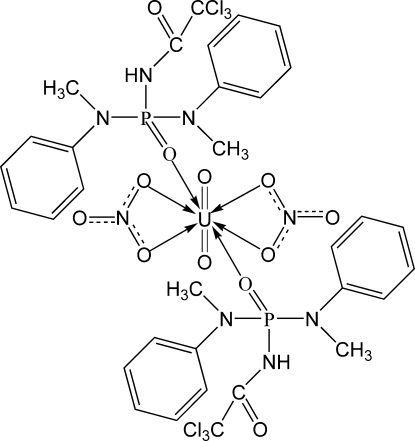


## Experimental

### 

#### Crystal data

[U(NO_3_)_2_O_2_(C_16_H_17_Cl_3_N_3_O_2_P)_2_]
*M* *_r_* = 1235.34
Monoclinic, 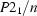

*a* = 10.2180 (4) Å
*b* = 16.2228 (6) Å
*c* = 28.4327 (7) Å
β = 97.421 (3)°
*V* = 4673.7 (3) Å^3^
*Z* = 4
Mo *K*α radiation
μ = 3.95 mm^−1^
*T* = 293 K
0.40 × 0.30 × 0.20 mm

#### Data collection

Oxford Diffraction Xcalibur3 diffractometer
Absorption correction: multi-scan (*CrysAlis RED*; Oxford Diffraction, 2006 ▶) *T*_min_ = 0.301, *T*_max_ = 0.506
24426 measured reflections
10643 independent reflections
7638 reflections with *I* > 2σ(*I*)
*R*_int_ = 0.041

#### Refinement

*R*[*F* ^2^ > 2σ(*F* ^2^)] = 0.048
*wR*(*F* ^2^) = 0.142
*S* = 0.97
10643 reflections
618 parameters
24 restraints
H-atom parameters constrained
Δρ_max_ = 1.39 e Å^−3^
Δρ_min_ = −1.02 e Å^−3^

### 

Data collection: *CrysAlis CCD* (Oxford Diffraction, 2006 ▶); cell refinement: *CrysAlis RED* (Oxford Diffraction, 2006 ▶); data reduction: *CrysAlis RED*; program(s) used to solve structure: *SHELXS97* (Sheldrick, 2008 ▶); program(s) used to refine structure: *SHELXL97* (Sheldrick, 2008 ▶); molecular graphics: *ORTEP-3 for Windows* (Farrugia, 1997 ▶); software used to prepare material for publication: *WinGX* (Farrugia, 1999 ▶).

## Supplementary Material

Crystal structure: contains datablocks I, global. DOI: 10.1107/S1600536810005611/cv2695sup1.cif

Structure factors: contains datablocks I. DOI: 10.1107/S1600536810005611/cv2695Isup2.hkl

Additional supplementary materials:  crystallographic information; 3D view; checkCIF report

## Figures and Tables

**Table 1:** Hydrogen-bond geometry (Å, °)

*D*—H⋯*A*	*D*—H	H⋯*A*	*D*⋯*A*	*D*—H⋯*A*
N11—H11*A*⋯O6	0.86	2.06	2.811 (6)	146
N21—H21*A*⋯O8	0.86	2.11	2.861 (6)	146

**Fig. 1.**
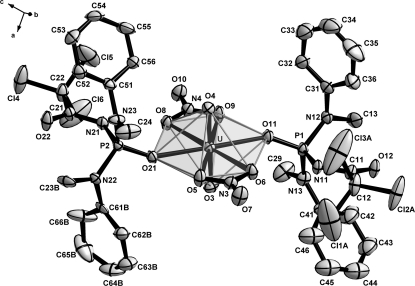
View of (I) with atomic numbering. Displacement ellipsoids are drawn at the 25% probability level. Only major parts of disordered atoms are shown. Hydrogen atoms omitted for clarity.

## supplementary crystallographic information

### Comment

Recently, we reported the synthesis and crystal structure of *N*-{bis[methyl(phenyl)amino]phosphoryl}-2,2,2-trichloroacetamide (*L*) (Znovjyak *et al.*, 2009). In our ongoing study of complexation properties of *L* we obtained the title compound, (I), and determined its crystal structure.

In (I) (Fig. 1), the coordination polyhedra of the uranium ions are slightly distorted hexagonal bipyramids (Keppert, 1982) where oxo ligands fill place of axial positions and two oxygen atoms of the phosphoryl groups from two *L* molecules and four oxygen atoms from two nitrate groups situated in the equatorial positions. The nitrate groups additionally form intramolecular hydrogen bonding with the hydrogen atoms of the N—H groups of the *L* ligands (Table 1). In the crystal structure of the complex, the phosphoryl group is situated in an *anti*-position with respect to the carbonyl group as in the crystal structure of the *L* which was described previously (Znovjyak *et al.*, 2009).

The U—O(oxo ligands) distances are equal to 1.750 (4) Å and 1.756 (4) Å. The U—O(NO_3_) and U—O(PO) distances fall in the range 2.515 (4)–2.564 (4) Å and 2.352 (4)–2.372 (4) Å, respectively and close to the corresponding values of the uranium complex with similar ligand previously published (Amirkhanov *et al.*, 1997). The coordinated nitrate groups have almost planar triangular structures. The O—N—O angles of the chelate rings (114.6 (5) and 115.1 (5)°) are shorter as compared to other angles O—N—O (121.9 (5)-123.0 (5)°) and bond lengths are slightly distorted: N—O(non-coordinated) and N—O(coordinated) are in the range of 1.198 (7) Å and 1.255 (7)–1.271 (6) Å, respectively. Bond lengths in the fragment C(O)NHP(O) are slightly changed upon complexation. The P—N bond distances between phosphorus atoms and nitrogen atoms of the amine substituents are shortened with respect to observed values in *L* (1.634 (4)–1.649 (4) Å) and fall in the range 1.621 (5)–1.635 (6) Å, that can be explained by increasing π-donor bonding in the (CH_3_N)P(O) fragment due to complexation, but this effect is weaken by π-acceptor influence of the phenyl group. The P—N(NH) and C—N distances do not change upon ligand coordination and are equal to 1.680 (5) Å, 1.683 (5) Å and 1.350 (7) Å, 1.358 (7) Å, respectively. In the non-coordinated molecule (*L*), the P—O bond lengths are 1.483 (3) Å, 1.478 (3) Å, while in (I) they are 1.473 (4) Å and 1.492 (4) Å, respectively.

The environment of the phosphorus atoms in the coordinated *L* has a slightly distorted tetrahedral geometry. The environment of the amide nitrogen atoms is practically planar and the sum of the adjacent bond angles is equal to 360° indicating sp^2^-hybridization.

### Experimental

The synthesis of *L* was carried out according to the procedure described previously (Znovjyak *et al.*, 2009).

Hydrated nitrate UO_2_(NO_3_)_2_2H_2_O (1 mmol) was solved upon heating in a methanol (10 ml). The solution was dehydrated by HC(OC_2_H_5_)_3_ (2 mmol), then heated to the boiling point and cooled down. The resulting solution was added to the solution of *L* (2 mmol) in isopropanol (10 ml) and was left in a vacuum desiccator over CaCl_2_ at room temperature. After 1 day, the yellow crystals were filtered off and washed with cold isopropanol and dried on the air (yield 85%). IR (KBr,cm^-1^): 3250 ν(NH), 1745 ν(CO), 1595, 1535, 1500, 1450 ν(CN), 1390, 1280, 1220, 1140 ν(PO), 1080, 1030, 950, 885, 820, 700.

### Refinement

All H atoms were placed at calculated positions and treated as riding on their parent atoms [C—H = 0.93 and 0.96 Å, and *U*_iso_(H) = 1.2 and 1.5*U*_eq_(C), N—H = 0.86 Å and *U*_iso_(H) = 1.2*U*_eq_(N)]. In one trichloromethyl group, atoms Cl1–Cl3 were treated as disordered between two orientations A and B, respectively, with the occupancies refined to 0.567 (15) and 0.433 (15). The methyl(phenyl)amino fragment (C23, C61–C66) was also treated as disordered over two conformations with the occupancies refined to 0.60 (4) and 0.40 (4), respectively.

### Crystal data

**Table:**

[U(NO_3_)2O_2_(C_16_H_17_Cl_3_N_3_O_2_P)_2_]	*F*(000) = 2408
*M**_r_* = 1235.34	*D*_x_ = 1.756 Mg m^−^^3^
Monoclinic, *P*2_1_/*n*	Mo *K*α radiation, λ = 0.71073 Å
*a* = 10.2180 (4) Å	Cell parameters from 37686 reflections
*b* = 16.2228 (6) Å	θ = 2.9–34.9°
*c* = 28.4327 (7) Å	µ = 3.95 mm^−^^1^
β = 97.421 (3)°	*T* = 293 K
*V* = 4673.7 (3) Å^3^	Block, yellow
*Z* = 4	0.40 × 0.30 × 0.20 mm

### Data collection

**Table:**

Oxford Diffraction Xcalibur3 diffractometer	10643 independent reflections
Radiation source: Enhance (Mo) X-ray Source	7638 reflections with *I* > 2σ(*I*)
graphite	*R*_int_ = 0.041
Detector resolution: 16.1827 pixels mm^-1^	θ_max_ = 27.5°, θ_min_ = 2.9°
ω scans	*h* = −13→13
Absorption correction: multi-scan (*CrysAlis RED*; Oxford Diffraction, 2006)	*k* = −21→8
*T*_min_ = 0.301, *T*_max_ = 0.506	*l* = −36→24
24426 measured reflections	

### Refinement

**Table:**

Refinement on *F*^2^	Primary atom site location: structure-invariant direct methods
Least-squares matrix: full	Secondary atom site location: difference Fourier map
*R*[*F*^2^ > 2σ(*F*^2^)] = 0.048	Hydrogen site location: inferred from neighbouring sites
*wR*(*F*^2^) = 0.142	H-atom parameters constrained
*S* = 0.97	*w* = 1/[σ^2^(*F*_o_^2^) + (0.0899*P*)^2^] where *P* = (*F*_o_^2^ + 2*F*_c_^2^)/3
10643 reflections	(Δ/σ)_max_ = 0.049
618 parameters	Δρ_max_ = 1.39 e Å^−^^3^
24 restraints	Δρ_min_ = −1.02 e Å^−^^3^

### Special details

**Table:**

Geometry. All esds (except the esd in the dihedral angle between two l.s. planes) are estimated using the full covariance matrix. The cell esds are taken into account individually in the estimation of esds in distances, angles and torsion angles; correlations between esds in cell parameters are only used when they are defined by crystal symmetry. An approximate (isotropic) treatment of cell esds is used for estimating esds involving l.s. planes.
Refinement. Refinement of *F*^2^ against ALL reflections. The weighted *R*-factor wR and goodness of fit *S* are based on *F*^2^, conventional *R*-factors *R* are based on *F*, with *F* set to zero for negative *F*^2^. The threshold expression of *F*^2^ > σ(*F*^2^) is used only for calculating *R*-factors(gt) etc. and is not relevant to the choice of reflections for refinement. *R*-factors based on *F*^2^ are statistically about twice as large as those based on *F*, and *R*- factors based on ALL data will be even larger.

### Fractional atomic coordinates and isotropic or equivalent isotropic displacement parameters (Å^2^)

**Table:**

	*x*	*y*	*z*	*U*_iso_*/*U*_eq_	Occ. (<1)
U	0.871559 (19)	0.121947 (13)	0.140066 (7)	0.04079 (9)	
P1	0.70900 (15)	0.16278 (10)	0.01657 (5)	0.0436 (3)	
P2	1.03225 (13)	0.08951 (9)	0.26388 (5)	0.0372 (3)	
O11	0.7295 (5)	0.1454 (3)	0.06851 (15)	0.0549 (11)	
O21	1.0084 (4)	0.0994 (3)	0.21199 (15)	0.0483 (10)	
N11	0.7594 (5)	0.2608 (3)	0.01177 (16)	0.0447 (11)	
H11A	0.8135	0.2798	0.0349	0.054*	
O8	0.8433 (4)	−0.0173 (3)	0.17804 (14)	0.0543 (11)	
N21	0.9692 (5)	−0.0036 (3)	0.27361 (17)	0.0444 (11)	
H21A	0.9185	−0.0256	0.2504	0.053*	
O10	0.7016 (6)	−0.1106 (3)	0.15164 (19)	0.0695 (15)	
O6	0.8993 (4)	0.2611 (3)	0.10364 (15)	0.0585 (12)	
O4	0.7473 (4)	0.1592 (3)	0.17174 (15)	0.0534 (10)	
O3	0.9941 (5)	0.0840 (3)	0.10814 (16)	0.0579 (11)	
O5	1.0157 (5)	0.2449 (3)	0.17055 (16)	0.0661 (13)	
N4	0.7533 (5)	−0.0451 (3)	0.14771 (18)	0.0487 (12)	
O7	1.0225 (7)	0.3611 (3)	0.1343 (2)	0.0839 (18)	
N22	1.1892 (4)	0.0966 (3)	0.28134 (17)	0.0434 (11)	
N23	0.9589 (5)	0.1539 (3)	0.2961 (2)	0.0563 (13)	
N13	0.7957 (6)	0.1049 (3)	−0.0149 (2)	0.0573 (14)	
O9	0.7204 (5)	0.0014 (3)	0.11269 (15)	0.0648 (13)	
N3	0.9823 (5)	0.2920 (3)	0.13605 (19)	0.0534 (13)	
N12	0.5536 (5)	0.1541 (4)	−0.00397 (17)	0.0520 (12)	
Cl5	0.7477 (3)	−0.1115 (2)	0.3015 (2)	0.196 (3)	
O12	0.6630 (5)	0.2949 (3)	−0.06185 (15)	0.0647 (13)	
Cl6	0.9530 (6)	−0.19012 (19)	0.26862 (15)	0.197 (2)	
O22	1.0626 (6)	−0.0249 (3)	0.34914 (16)	0.0727 (15)	
C51	0.8482 (6)	0.1325 (4)	0.3194 (2)	0.0497 (14)	
C21	0.9902 (6)	−0.0475 (4)	0.3143 (2)	0.0477 (14)	
C52	0.8613 (8)	0.1282 (4)	0.3677 (3)	0.0658 (19)	
H52A	0.9421	0.1393	0.3856	0.079*	
C41	0.8742 (8)	0.1335 (4)	−0.0495 (3)	0.0611 (18)	
C11	0.7230 (7)	0.3129 (4)	−0.0250 (2)	0.0495 (14)	
C31	0.4569 (7)	0.1903 (5)	0.0203 (3)	0.0636 (18)	
C56	0.7273 (7)	0.1217 (6)	0.2935 (3)	0.077 (3)	
H56A	0.7162	0.1285	0.2608	0.092*	
C53	0.7531 (10)	0.1072 (6)	0.3897 (3)	0.085 (3)	
H53A	0.7622	0.1034	0.4226	0.102*	
C22	0.9179 (9)	−0.1300 (4)	0.3144 (3)	0.067 (2)	
C54	0.6373 (8)	0.0924 (8)	0.3650 (3)	0.094 (3)	
H54A	0.5662	0.0766	0.3803	0.113*	
C32	0.4102 (9)	0.1501 (7)	0.0586 (3)	0.082 (2)	
H32A	0.4463	0.0999	0.0695	0.099*	
C36	0.4023 (9)	0.2673 (6)	0.0069 (3)	0.091 (3)	
H36A	0.4341	0.2974	−0.0170	0.110*	
C12	0.7731 (5)	0.3968 (3)	−0.01573 (12)	0.084 (3)	
C24	0.9943 (10)	0.2414 (5)	0.2964 (4)	0.107 (4)	
H24A	0.9431	0.2710	0.3169	0.160*	
H24B	0.9767	0.2630	0.2648	0.160*	
H24C	1.0865	0.2475	0.3077	0.160*	
C42	0.8354 (15)	0.1152 (7)	−0.0970 (4)	0.121 (3)	
H42A	0.7592	0.0846	−0.1059	0.145*	
C29	0.7946 (12)	0.0162 (5)	−0.0064 (4)	0.106 (4)	
H29A	0.8488	−0.0109	−0.0268	0.159*	
H29B	0.7058	−0.0040	−0.0128	0.159*	
H29C	0.8285	0.0052	0.0261	0.159*	
C55	0.6225 (8)	0.1005 (7)	0.3167 (4)	0.099 (3)	
H55A	0.5402	0.0915	0.2993	0.119*	
C46	0.9891 (12)	0.1742 (7)	−0.0377 (5)	0.127 (3)	
H46A	1.0155	0.1856	−0.0058	0.153*	
C35	0.3010 (14)	0.2981 (10)	0.0294 (6)	0.154 (6)	
H35A	0.2633	0.3484	0.0196	0.184*	
Cl4	0.9432 (4)	−0.1782 (2)	0.36751 (10)	0.1564 (17)	
C43	0.9124 (14)	0.1433 (8)	−0.1318 (4)	0.121 (3)	
H43A	0.8862	0.1332	−0.1639	0.145*	
C44	1.0247 (13)	0.1852 (7)	−0.1174 (6)	0.127 (3)	
H44A	1.0737	0.2051	−0.1404	0.153*	
C45	1.0703 (13)	0.1998 (7)	−0.0707 (5)	0.127 (3)	
H45A	1.1509	0.2255	−0.0615	0.153*	
C13	0.5054 (8)	0.1276 (6)	−0.0522 (3)	0.082 (3)	
H13A	0.4107	0.1271	−0.0565	0.124*	
H13B	0.5377	0.0733	−0.0574	0.124*	
H13C	0.5361	0.1652	−0.0745	0.124*	
C33	0.3089 (13)	0.1862 (11)	0.0799 (4)	0.119 (4)	
H33A	0.2780	0.1597	0.1053	0.143*	
C34	0.2554 (13)	0.2578 (14)	0.0648 (5)	0.142 (6)	
H34A	0.1860	0.2798	0.0789	0.170*	
C61A	1.277 (3)	0.0532 (18)	0.2554 (12)	0.035 (7)	0.40 (4)
C62A	1.325 (3)	0.084 (2)	0.2186 (12)	0.080 (8)	0.40 (4)
H62A	1.2954	0.1361	0.2082	0.096*	0.40 (4)
C63A	1.414 (3)	0.048 (2)	0.1948 (11)	0.080 (8)	0.40 (4)
H63A	1.4456	0.0742	0.1694	0.096*	0.40 (4)
C64A	1.454 (5)	−0.026 (3)	0.2086 (11)	0.119 (19)	0.40 (4)
H64A	1.5097	−0.0551	0.1908	0.143*	0.40 (4)
C65A	1.415 (2)	−0.0641 (16)	0.2503 (14)	0.076 (9)	0.40 (4)
H65A	1.4524	−0.1133	0.2624	0.092*	0.40 (4)
C66A	1.318 (3)	−0.0223 (15)	0.2719 (9)	0.050 (6)	0.40 (4)
H66A	1.2823	−0.0462	0.2971	0.060*	0.40 (4)
C23A	1.235 (4)	0.1244 (19)	0.3328 (12)	0.045 (7)	0.40 (4)
H23A	1.3295	0.1259	0.3382	0.068*	0.40 (4)
H23B	1.2027	0.0864	0.3544	0.068*	0.40 (4)
H23C	1.2006	0.1785	0.3376	0.068*	0.40 (4)
C62B	1.3294 (17)	0.1168 (16)	0.2199 (7)	0.067 (5)	0.60 (4)
H62B	1.3008	0.1712	0.2190	0.080*	0.60 (4)
C61B	1.280 (3)	0.0668 (16)	0.2495 (10)	0.057 (8)	0.60 (4)
C63B	1.417 (2)	0.096 (3)	0.1911 (7)	0.108 (9)	0.60 (4)
H63B	1.4534	0.1352	0.1728	0.129*	0.60 (4)
C64B	1.452 (2)	0.018 (3)	0.1893 (14)	0.123 (14)	0.60 (4)
H64B	1.4957	−0.0013	0.1648	0.147*	0.60 (4)
C65B	1.421 (3)	−0.038 (3)	0.2255 (18)	0.157 (17)	0.60 (4)
H65B	1.4650	−0.0879	0.2312	0.188*	0.60 (4)
C66B	1.320 (3)	−0.0131 (16)	0.2518 (17)	0.137 (14)	0.60 (4)
H66B	1.2815	−0.0508	0.2704	0.164*	0.60 (4)
C23B	1.257 (3)	0.116 (2)	0.3260 (10)	0.086 (11)	0.60 (4)
H23D	1.3502	0.1150	0.3246	0.129*	0.60 (4)
H23E	1.2352	0.0767	0.3489	0.129*	0.60 (4)
H23F	1.2316	0.1703	0.3351	0.129*	0.60 (4)
Cl1A	0.9423 (8)	0.4061 (6)	0.0048 (7)	0.310 (13)	0.567 (15)
Cl2A	0.7365 (18)	0.4709 (5)	−0.0607 (3)	0.168 (6)	0.567 (15)
Cl3A	0.701 (2)	0.4297 (7)	0.0339 (4)	0.312 (13)	0.567 (15)
Cl1B	0.9345 (8)	0.3982 (7)	−0.0312 (7)	0.159 (6)	0.433 (15)
Cl2B	0.6768 (16)	0.4636 (6)	−0.0549 (5)	0.121 (5)	0.433 (15)
Cl3B	0.7851 (17)	0.4390 (6)	0.04167 (19)	0.145 (5)	0.433 (15)

### Atomic displacement parameters (Å^2^)

**Table:**

	*U*^11^	*U*^22^	*U*^33^	*U*^12^	*U*^13^	*U*^23^
U	0.03685 (13)	0.04673 (14)	0.03773 (13)	−0.00300 (9)	0.00076 (8)	0.00362 (9)
P1	0.0466 (8)	0.0454 (8)	0.0363 (7)	−0.0095 (7)	−0.0034 (6)	0.0026 (6)
P2	0.0280 (6)	0.0385 (7)	0.0443 (8)	−0.0023 (6)	0.0012 (5)	0.0020 (6)
O11	0.055 (3)	0.064 (3)	0.042 (2)	−0.014 (2)	−0.0080 (19)	0.008 (2)
O21	0.039 (2)	0.053 (2)	0.049 (2)	−0.0072 (18)	−0.0080 (18)	0.0098 (19)
N11	0.054 (3)	0.046 (3)	0.032 (2)	−0.004 (2)	−0.006 (2)	0.005 (2)
O8	0.058 (3)	0.057 (2)	0.045 (2)	−0.013 (2)	−0.005 (2)	0.003 (2)
N21	0.045 (3)	0.041 (3)	0.044 (3)	−0.012 (2)	−0.004 (2)	0.003 (2)
O10	0.083 (4)	0.059 (3)	0.064 (3)	−0.029 (3)	0.000 (3)	0.005 (2)
O6	0.062 (3)	0.052 (3)	0.056 (3)	−0.016 (2)	−0.013 (2)	0.007 (2)
O4	0.049 (2)	0.059 (3)	0.053 (3)	0.009 (2)	0.0075 (19)	0.007 (2)
O3	0.059 (3)	0.058 (3)	0.060 (3)	0.004 (2)	0.022 (2)	0.000 (2)
O5	0.077 (3)	0.061 (3)	0.054 (3)	−0.016 (2)	−0.016 (2)	0.011 (2)
N4	0.053 (3)	0.049 (3)	0.043 (3)	−0.013 (2)	0.002 (2)	−0.001 (2)
O7	0.109 (5)	0.060 (3)	0.078 (4)	−0.034 (3)	−0.006 (3)	0.004 (3)
N22	0.032 (2)	0.056 (3)	0.041 (3)	−0.005 (2)	−0.0008 (19)	−0.001 (2)
N23	0.051 (3)	0.043 (3)	0.079 (4)	0.002 (2)	0.025 (3)	−0.002 (3)
N13	0.064 (4)	0.047 (3)	0.060 (3)	−0.008 (3)	0.005 (3)	0.001 (2)
O9	0.073 (3)	0.065 (3)	0.049 (3)	−0.021 (2)	−0.018 (2)	0.008 (2)
N3	0.055 (3)	0.053 (3)	0.052 (3)	−0.013 (3)	0.002 (2)	0.001 (3)
N12	0.047 (3)	0.066 (3)	0.040 (3)	−0.008 (3)	−0.003 (2)	0.003 (3)
Cl5	0.086 (2)	0.150 (3)	0.336 (7)	−0.052 (2)	−0.036 (3)	0.115 (4)
O12	0.089 (4)	0.063 (3)	0.036 (2)	−0.007 (3)	−0.014 (2)	0.005 (2)
Cl6	0.366 (7)	0.0752 (18)	0.177 (3)	−0.072 (3)	0.140 (4)	−0.052 (2)
O22	0.094 (4)	0.068 (3)	0.048 (3)	−0.018 (3)	−0.023 (3)	0.004 (2)
C51	0.041 (3)	0.055 (4)	0.054 (4)	0.007 (3)	0.009 (3)	−0.007 (3)
C21	0.048 (3)	0.046 (3)	0.047 (3)	−0.003 (3)	−0.006 (3)	0.004 (3)
C52	0.059 (4)	0.078 (5)	0.059 (4)	−0.004 (4)	0.000 (3)	−0.006 (4)
C41	0.059 (4)	0.051 (4)	0.075 (5)	0.009 (3)	0.017 (4)	−0.001 (3)
C11	0.063 (4)	0.051 (3)	0.034 (3)	−0.002 (3)	0.004 (3)	0.004 (3)
C31	0.048 (4)	0.080 (5)	0.060 (4)	−0.012 (4)	−0.004 (3)	0.002 (4)
C56	0.051 (4)	0.134 (8)	0.046 (4)	0.008 (4)	0.008 (3)	0.007 (4)
C53	0.091 (7)	0.115 (7)	0.052 (4)	0.006 (6)	0.025 (4)	−0.003 (4)
C22	0.086 (5)	0.052 (4)	0.059 (4)	−0.015 (4)	−0.008 (4)	0.013 (3)
C54	0.055 (5)	0.149 (9)	0.087 (7)	−0.014 (5)	0.039 (5)	−0.006 (6)
C32	0.066 (5)	0.120 (7)	0.061 (5)	−0.018 (5)	0.010 (4)	0.006 (5)
C36	0.086 (6)	0.100 (7)	0.089 (6)	0.030 (5)	0.014 (5)	0.020 (5)
C12	0.139 (9)	0.051 (4)	0.053 (4)	0.002 (5)	−0.013 (5)	0.011 (3)
C24	0.110 (8)	0.048 (4)	0.178 (11)	−0.012 (5)	0.077 (8)	−0.016 (5)
C42	0.141 (8)	0.146 (7)	0.083 (5)	0.016 (6)	0.046 (5)	0.001 (5)
C29	0.160 (10)	0.055 (5)	0.112 (8)	−0.009 (6)	0.052 (7)	−0.008 (5)
C55	0.039 (4)	0.160 (9)	0.099 (7)	−0.011 (5)	0.009 (4)	−0.028 (7)
C46	0.109 (5)	0.101 (4)	0.184 (8)	−0.005 (4)	0.065 (5)	−0.009 (5)
C35	0.110 (10)	0.156 (13)	0.188 (15)	0.064 (10)	−0.005 (10)	−0.032 (12)
Cl4	0.230 (4)	0.127 (2)	0.0944 (19)	−0.088 (3)	−0.047 (2)	0.0574 (18)
C43	0.141 (8)	0.146 (7)	0.083 (5)	0.016 (6)	0.046 (5)	0.001 (5)
C44	0.109 (5)	0.101 (4)	0.184 (8)	−0.005 (4)	0.065 (5)	−0.009 (5)
C45	0.109 (5)	0.101 (4)	0.184 (8)	−0.005 (4)	0.065 (5)	−0.009 (5)
C13	0.060 (4)	0.135 (8)	0.049 (4)	−0.030 (5)	−0.008 (3)	−0.021 (4)
C33	0.098 (9)	0.181 (14)	0.082 (7)	−0.012 (9)	0.026 (6)	−0.001 (8)
C34	0.086 (9)	0.24 (2)	0.101 (10)	−0.004 (11)	0.031 (7)	−0.045 (12)
C61A	0.022 (12)	0.046 (13)	0.036 (11)	0.006 (9)	−0.003 (8)	−0.003 (9)
C62A	0.109 (19)	0.069 (16)	0.069 (12)	0.028 (13)	0.038 (11)	0.016 (12)
C63A	0.109 (19)	0.069 (16)	0.069 (12)	0.028 (13)	0.038 (11)	0.016 (12)
C64A	0.20 (5)	0.12 (3)	0.053 (18)	0.01 (3)	0.07 (2)	−0.020 (18)
C65A	0.052 (12)	0.047 (12)	0.13 (2)	0.036 (9)	0.000 (13)	0.013 (13)
C66A	0.046 (12)	0.051 (12)	0.056 (12)	0.005 (9)	0.018 (8)	−0.001 (8)
C23A	0.051 (17)	0.058 (12)	0.026 (10)	−0.010 (10)	0.001 (10)	0.008 (10)
C62B	0.052 (8)	0.087 (12)	0.063 (9)	−0.002 (8)	0.016 (6)	0.004 (9)
C61B	0.038 (11)	0.062 (11)	0.071 (17)	−0.001 (8)	0.005 (10)	−0.012 (10)
C63B	0.083 (12)	0.16 (3)	0.087 (13)	0.008 (16)	0.042 (10)	0.007 (16)
C64B	0.083 (14)	0.15 (3)	0.15 (3)	−0.001 (16)	0.065 (16)	−0.05 (2)
C65B	0.15 (3)	0.12 (3)	0.19 (5)	0.08 (2)	−0.02 (3)	−0.03 (2)
C66B	0.073 (15)	0.096 (18)	0.26 (4)	−0.003 (13)	0.08 (2)	−0.03 (2)
C23B	0.035 (8)	0.17 (3)	0.052 (12)	−0.018 (10)	0.004 (7)	−0.048 (11)
Cl1A	0.332 (17)	0.126 (7)	0.40 (2)	−0.145 (9)	−0.229 (17)	0.104 (11)
Cl2A	0.360 (18)	0.075 (4)	0.057 (3)	−0.049 (6)	−0.020 (6)	0.021 (3)
Cl3A	0.77 (4)	0.076 (5)	0.104 (6)	−0.019 (13)	0.097 (13)	−0.050 (4)
Cl1B	0.113 (7)	0.113 (6)	0.266 (15)	−0.065 (6)	0.077 (8)	−0.060 (8)
Cl2B	0.192 (10)	0.045 (4)	0.112 (7)	0.015 (5)	−0.040 (6)	0.034 (3)
Cl3B	0.334 (15)	0.059 (4)	0.034 (3)	−0.061 (6)	−0.007 (5)	−0.008 (3)

### Geometric parameters (Å, °)

**Table:**

U—O3	1.750 (4)	C36—C35	1.379 (16)
U—O4	1.756 (4)	C36—H36A	0.9300
U—O21	2.352 (4)	C12—Cl1A	1.7587 (10)
U—O11	2.372 (4)	C12—Cl2A	1.7590 (10)
U—O6	2.515 (4)	C12—Cl3B	1.7591 (10)
U—O8	2.537 (4)	C12—Cl2B	1.7593 (10)
U—O9	2.550 (4)	C12—Cl3A	1.7600 (10)
U—O5	2.564 (4)	C12—Cl1B	1.7606 (10)
U—N4	2.986 (5)	C24—H24A	0.9600
U—N3	2.989 (5)	C24—H24B	0.9600
P1—O11	1.492 (4)	C24—H24C	0.9600
P1—N12	1.626 (5)	C42—C43	1.418 (16)
P1—N13	1.634 (6)	C42—H42A	0.9300
P1—N11	1.683 (5)	C29—H29A	0.9600
P2—O21	1.473 (4)	C29—H29B	0.9600
P2—N22	1.621 (5)	C29—H29C	0.9600
P2—N23	1.635 (6)	C55—H55A	0.9300
P2—N21	1.680 (5)	C46—C45	1.395 (15)
N11—C11	1.358 (7)	C46—H46A	0.9300
N11—H11A	0.8600	C35—C34	1.33 (2)
O8—N4	1.260 (6)	C35—H35A	0.9300
N21—C21	1.350 (7)	C43—C44	1.351 (17)
N21—H21A	0.8600	C43—H43A	0.9300
O10—N4	1.198 (6)	C44—C45	1.371 (17)
O6—N3	1.271 (6)	C44—H44A	0.9300
O5—N3	1.255 (7)	C45—H45A	0.9300
N4—O9	1.260 (6)	C13—H13A	0.9600
O7—N3	1.198 (7)	C13—H13B	0.9600
N22—C23B	1.40 (3)	C13—H13C	0.9600
N22—C61A	1.42 (2)	C33—C34	1.33 (2)
N22—C61B	1.46 (2)	C33—H33A	0.9300
N22—C23A	1.54 (4)	C34—H34A	0.9300
N23—C51	1.424 (8)	C61A—C62A	1.315 (19)
N23—C24	1.464 (9)	C61A—C66A	1.36 (2)
N13—C41	1.425 (10)	C62A—C63A	1.33 (2)
N13—C29	1.459 (10)	C62A—H62A	0.9300
N12—C31	1.405 (9)	C63A—C64A	1.32 (4)
N12—C13	1.461 (8)	C63A—H63A	0.9300
Cl5—C22	1.756 (9)	C64A—C65A	1.44 (4)
O12—C11	1.180 (7)	C64A—H64A	0.9300
Cl6—C22	1.702 (9)	C65A—C66A	1.41 (3)
O22—C21	1.215 (7)	C65A—H65A	0.9300
C51—C56	1.365 (10)	C66A—H66A	0.9300
C51—C52	1.365 (10)	C23A—H23A	0.9600
C21—C22	1.530 (9)	C23A—H23B	0.9600
C52—C53	1.381 (12)	C23A—H23C	0.9600
C52—H52A	0.9300	C62B—C63B	1.336 (17)
C41—C46	1.351 (14)	C62B—C61B	1.315 (18)
C41—C42	1.390 (15)	C62B—H62B	0.9300
C11—C12	1.467 (8)	C61B—C66B	1.360 (19)
C31—C36	1.400 (11)	C63B—C64B	1.32 (3)
C31—C32	1.403 (11)	C63B—H63B	0.9300
C56—C55	1.371 (11)	C64B—C65B	1.43 (4)
C56—H56A	0.9300	C64B—H64B	0.9300
C53—C54	1.317 (12)	C65B—C66B	1.41 (2)
C53—H53A	0.9300	C65B—H65B	0.9300
C22—Cl4	1.690 (7)	C66B—H66B	0.9300
C54—C55	1.371 (12)	C23B—H23D	0.9600
C54—H54A	0.9300	C23B—H23E	0.9600
C32—C33	1.395 (15)	C23B—H23F	0.9600
C32—H32A	0.9300		
			
O3—U—O4	179.3 (2)	C21—C22—Cl5	108.4 (5)
O3—U—O21	90.65 (19)	Cl4—C22—Cl5	107.5 (5)
O4—U—O21	89.76 (17)	Cl6—C22—Cl5	103.8 (5)
O3—U—O11	90.6 (2)	C53—C54—C55	119.4 (8)
O4—U—O11	88.96 (18)	C53—C54—H54A	120.3
O21—U—O11	178.71 (15)	C55—C54—H54A	120.3
O3—U—O6	88.62 (19)	C33—C32—C31	119.1 (10)
O4—U—O6	91.73 (18)	C33—C32—H32A	120.4
O21—U—O6	114.30 (13)	C31—C32—H32A	120.4
O11—U—O6	65.94 (14)	C35—C36—C31	119.6 (11)
O3—U—O8	92.05 (19)	C35—C36—H36A	120.2
O4—U—O8	87.61 (18)	C31—C36—H36A	120.2
O21—U—O8	65.11 (13)	C11—C12—Cl1A	116.4 (5)
O11—U—O8	114.64 (14)	C11—C12—Cl2A	117.9 (5)
O6—U—O8	179.10 (14)	Cl1A—C12—Cl2A	106.9 (6)
O3—U—O9	91.0 (2)	C11—C12—Cl3B	120.6 (5)
O4—U—O9	88.3 (2)	Cl1A—C12—Cl3B	73.2 (7)
O21—U—O9	114.52 (14)	Cl2A—C12—Cl3B	113.0 (6)
O11—U—O9	65.26 (15)	C11—C12—Cl2B	107.7 (5)
O6—U—O9	131.19 (13)	Cl1A—C12—Cl2B	127.1 (7)
O8—U—O9	49.41 (13)	Cl2A—C12—Cl2B	21.7 (9)
O3—U—O5	91.8 (2)	Cl3B—C12—Cl2B	108.3 (7)
O4—U—O5	88.9 (2)	C11—C12—Cl3A	104.7 (6)
O21—U—O5	64.90 (14)	Cl1A—C12—Cl3A	101.8 (8)
O11—U—O5	115.25 (15)	Cl2A—C12—Cl3A	107.6 (7)
O6—U—O5	49.47 (13)	Cl3B—C12—Cl3A	28.9 (8)
O8—U—O5	129.89 (14)	Cl2B—C12—Cl3A	93.5 (7)
O9—U—O5	177.16 (17)	C11—C12—Cl1B	106.4 (5)
O3—U—N4	92.39 (19)	Cl1A—C12—Cl1B	33.9 (6)
O4—U—N4	87.04 (18)	Cl2A—C12—Cl1B	85.9 (7)
O21—U—N4	89.79 (14)	Cl3B—C12—Cl1B	105.7 (7)
O11—U—N4	89.96 (14)	Cl2B—C12—Cl1B	107.4 (8)
O6—U—N4	155.89 (13)	Cl3A—C12—Cl1B	134.5 (8)
O8—U—N4	24.69 (13)	N23—C24—H24A	109.5
O9—U—N4	24.74 (13)	N23—C24—H24B	109.5
O5—U—N4	154.40 (14)	H24A—C24—H24B	109.5
O3—U—N3	90.31 (19)	N23—C24—H24C	109.5
O4—U—N3	90.27 (19)	H24A—C24—H24C	109.5
O21—U—N3	89.49 (14)	H24B—C24—H24C	109.5
O11—U—N3	90.70 (15)	C41—C42—C43	119.7 (13)
O6—U—N3	24.85 (13)	C41—C42—H42A	120.1
O8—U—N3	154.51 (14)	C43—C42—H42A	120.1
O9—U—N3	155.93 (14)	N13—C29—H29A	109.5
O5—U—N3	24.62 (13)	N13—C29—H29B	109.5
N4—U—N3	177.22 (14)	H29A—C29—H29B	109.5
O11—P1—N12	110.2 (3)	N13—C29—H29C	109.5
O11—P1—N13	114.6 (3)	H29A—C29—H29C	109.5
N12—P1—N13	109.1 (3)	H29B—C29—H29C	109.5
O11—P1—N11	104.7 (2)	C54—C55—C56	120.9 (8)
N12—P1—N11	110.3 (3)	C54—C55—H55A	119.5
N13—P1—N11	107.8 (3)	C56—C55—H55A	119.5
O21—P2—N22	109.1 (3)	C41—C46—C45	123.5 (14)
O21—P2—N23	117.6 (3)	C41—C46—H46A	118.3
N22—P2—N23	106.9 (3)	C45—C46—H46A	118.3
O21—P2—N21	104.2 (2)	C34—C35—C36	122.0 (14)
N22—P2—N21	113.5 (3)	C34—C35—H35A	119.0
N23—P2—N21	105.6 (3)	C36—C35—H35A	119.0
P1—O11—U	150.6 (3)	C44—C43—C42	118.4 (13)
P2—O21—U	152.9 (3)	C44—C43—H43A	120.8
C11—N11—P1	126.4 (4)	C42—C43—H43A	120.8
C11—N11—H11A	116.8	C43—C44—C45	123.4 (13)
P1—N11—H11A	116.8	C43—C44—H44A	118.3
N4—O8—U	98.1 (3)	C45—C44—H44A	118.3
C21—N21—P2	126.3 (4)	C44—C45—C46	116.4 (13)
C21—N21—H21A	116.8	C44—C45—H45A	121.8
P2—N21—H21A	116.8	C46—C45—H45A	121.8
N3—O6—U	98.9 (3)	N12—C13—H13A	109.5
N3—O5—U	97.0 (3)	N12—C13—H13B	109.5
O10—N4—O8	123.0 (5)	H13A—C13—H13B	109.5
O10—N4—O9	121.9 (5)	N12—C13—H13C	109.5
O8—N4—O9	115.1 (5)	H13A—C13—H13C	109.5
O10—N4—U	177.2 (5)	H13B—C13—H13C	109.5
O8—N4—U	57.3 (3)	C34—C33—C32	121.6 (13)
O9—N4—U	57.9 (3)	C34—C33—H33A	119.2
C23B—N22—C61A	108 (2)	C32—C33—H33A	119.2
C23B—N22—C61B	111.1 (17)	C33—C34—C35	120.1 (14)
C61A—N22—C61B	11.1 (17)	C33—C34—H34A	119.9
C23B—N22—C23A	12 (2)	C35—C34—H34A	119.9
C61A—N22—C23A	120 (2)	C62A—C61A—C66A	119.2 (18)
C61B—N22—C23A	123.2 (19)	C62A—C61A—N22	124 (2)
C23B—N22—P2	130.2 (13)	C66A—C61A—N22	117 (2)
C61A—N22—P2	118.2 (16)	C63A—C62A—C61A	126 (2)
C61B—N22—P2	118.0 (13)	C63A—C62A—H62A	117.1
C23A—N22—P2	118.4 (16)	C61A—C62A—H62A	117.0
C51—N23—C24	116.4 (6)	C64A—C63A—C62A	117 (2)
C51—N23—P2	123.6 (4)	C64A—C63A—H63A	121.3
C24—N23—P2	119.4 (5)	C62A—C63A—H63A	121.3
C41—N13—C29	116.9 (7)	C63A—C64A—C65A	122 (2)
C41—N13—P1	125.7 (4)	C63A—C64A—H64A	119.1
C29—N13—P1	117.4 (6)	C65A—C64A—H64A	119.2
N4—O9—U	97.4 (3)	C66A—C65A—C64A	116 (2)
O7—N3—O5	122.8 (6)	C66A—C65A—H65A	121.9
O7—N3—O6	122.6 (6)	C64A—C65A—H65A	121.9
O5—N3—O6	114.6 (5)	C61A—C66A—C65A	119 (2)
O7—N3—U	177.9 (5)	C61A—C66A—H66A	120.4
O5—N3—U	58.3 (3)	C65A—C66A—H66A	120.4
O6—N3—U	56.2 (3)	N22—C23A—H23A	109.5
C31—N12—C13	114.3 (6)	N22—C23A—H23B	109.5
C31—N12—P1	120.2 (4)	H23A—C23A—H23B	109.5
C13—N12—P1	123.9 (5)	N22—C23A—H23C	109.5
C56—C51—C52	120.0 (7)	H23A—C23A—H23C	109.5
C56—C51—N23	119.9 (6)	H23B—C23A—H23C	109.5
C52—C51—N23	119.9 (6)	C63B—C62B—C61B	125.5 (18)
O22—C21—N21	123.6 (6)	C63B—C62B—H62B	117.3
O22—C21—C22	120.3 (6)	C61B—C62B—H62B	117.3
N21—C21—C22	116.1 (5)	C62B—C61B—C66B	118.6 (16)
C51—C52—C53	119.2 (7)	C62B—C61B—N22	121.4 (18)
C51—C52—H52A	120.4	C66B—C61B—N22	119.9 (18)
C53—C52—H52A	120.4	C64B—C63B—C62B	118 (2)
C46—C41—C42	118.4 (10)	C64B—C63B—H63B	120.9
C46—C41—N13	122.4 (9)	C62B—C63B—H63B	120.9
C42—C41—N13	119.1 (8)	C63B—C64B—C65B	120 (2)
O12—C11—N11	126.1 (6)	C63B—C64B—H64B	120.2
O12—C11—C12	121.3 (5)	C65B—C64B—H64B	120.2
N11—C11—C12	112.6 (5)	C66B—C65B—C64B	116 (2)
C36—C31—C32	117.6 (8)	C66B—C65B—H65B	121.9
C36—C31—N12	121.3 (7)	C64B—C65B—H65B	121.9
C32—C31—N12	121.1 (7)	C61B—C66B—C65B	119 (2)
C51—C56—C55	118.8 (7)	C61B—C66B—H66B	120.5
C51—C56—H56A	120.6	C65B—C66B—H66B	120.4
C55—C56—H56A	120.6	N22—C23B—H23D	109.5
C54—C53—C52	121.4 (8)	N22—C23B—H23E	109.5
C54—C53—H53A	119.3	H23D—C23B—H23E	109.5
C52—C53—H53A	119.3	N22—C23B—H23F	109.5
C21—C22—Cl4	112.9 (5)	H23D—C23B—H23F	109.5
C21—C22—Cl6	110.5 (6)	H23E—C23B—H23F	109.5
Cl4—C22—Cl6	113.3 (5)		
			
N12—P1—O11—U	171.1 (5)	O4—U—N3—O7	−36 (12)
N13—P1—O11—U	47.7 (7)	O21—U—N3—O7	−126 (12)
N11—P1—O11—U	−70.2 (6)	O11—U—N3—O7	53 (12)
O3—U—O11—P1	−26.7 (6)	O6—U—N3—O7	57 (12)
O4—U—O11—P1	153.9 (6)	O8—U—N3—O7	−121 (12)
O21—U—O11—P1	162 (6)	O9—U—N3—O7	50 (13)
O6—U—O11—P1	61.5 (6)	O5—U—N3—O7	−123 (13)
O8—U—O11—P1	−119.2 (6)	N4—U—N3—O7	−51 (14)
O9—U—O11—P1	−117.5 (6)	O3—U—N3—O5	−93.6 (4)
O5—U—O11—P1	65.6 (6)	O4—U—N3—O5	86.8 (4)
N4—U—O11—P1	−119.1 (6)	O21—U—N3—O5	−2.9 (4)
N3—U—O11—P1	63.6 (6)	O11—U—N3—O5	175.8 (4)
N22—P2—O21—U	−166.1 (5)	O6—U—N3—O5	−179.6 (6)
N23—P2—O21—U	−44.2 (7)	O8—U—N3—O5	1.8 (6)
N21—P2—O21—U	72.3 (6)	O9—U—N3—O5	173.2 (4)
O3—U—O21—P2	−162.8 (6)	N4—U—N3—O5	72 (3)
O4—U—O21—P2	16.7 (6)	O3—U—N3—O6	86.1 (4)
O11—U—O21—P2	8(7)	O4—U—N3—O6	−93.5 (4)
O6—U—O21—P2	108.4 (6)	O21—U—N3—O6	176.7 (4)
O8—U—O21—P2	−70.8 (6)	O11—U—N3—O6	−4.6 (4)
O9—U—O21—P2	−71.4 (6)	O8—U—N3—O6	−178.5 (4)
O5—U—O21—P2	105.6 (6)	O9—U—N3—O6	−7.1 (6)
N4—U—O21—P2	−70.4 (6)	O5—U—N3—O6	179.6 (6)
N3—U—O21—P2	106.9 (6)	N4—U—N3—O6	−108 (3)
O11—P1—N11—C11	−157.5 (5)	O11—P1—N12—C31	46.0 (6)
N12—P1—N11—C11	−39.0 (6)	N13—P1—N12—C31	172.6 (5)
N13—P1—N11—C11	80.1 (6)	N11—P1—N12—C31	−69.2 (6)
O3—U—O8—N4	−91.3 (4)	O11—P1—N12—C13	−149.1 (6)
O4—U—O8—N4	88.1 (4)	N13—P1—N12—C13	−22.5 (7)
O21—U—O8—N4	179.0 (4)	N11—P1—N12—C13	95.7 (6)
O11—U—O8—N4	0.4 (4)	C24—N23—C51—C56	98.3 (9)
O6—U—O8—N4	131 (9)	P2—N23—C51—C56	−72.9 (8)
O9—U—O8—N4	−1.7 (3)	C24—N23—C51—C52	−77.4 (9)
O5—U—O8—N4	174.7 (3)	P2—N23—C51—C52	111.4 (7)
N3—U—O8—N4	173.7 (3)	P2—N21—C21—O22	−2.3 (10)
O21—P2—N21—C21	165.7 (5)	P2—N21—C21—C22	178.4 (5)
N22—P2—N21—C21	47.1 (6)	C56—C51—C52—C53	4.4 (11)
N23—P2—N21—C21	−69.7 (6)	N23—C51—C52—C53	−179.8 (7)
O3—U—O6—N3	−93.7 (4)	C29—N13—C41—C46	−107.2 (10)
O4—U—O6—N3	86.9 (4)	P1—N13—C41—C46	71.8 (10)
O21—U—O6—N3	−3.6 (4)	C29—N13—C41—C42	69.2 (11)
O11—U—O6—N3	175.0 (4)	P1—N13—C41—C42	−111.9 (8)
O8—U—O6—N3	45 (9)	P1—N11—C11—O12	−9.6 (10)
O9—U—O6—N3	176.1 (3)	P1—N11—C11—C12	173.6 (4)
O5—U—O6—N3	−0.2 (3)	C13—N12—C31—C36	−68.8 (9)
N4—U—O6—N3	173.5 (4)	P1—N12—C31—C36	97.5 (8)
O3—U—O5—N3	86.9 (4)	C13—N12—C31—C32	110.2 (8)
O4—U—O5—N3	−93.0 (4)	P1—N12—C31—C32	−83.5 (8)
O21—U—O5—N3	176.8 (4)	C52—C51—C56—C55	−4.8 (12)
O11—U—O5—N3	−4.7 (4)	N23—C51—C56—C55	179.5 (8)
O6—U—O5—N3	0.2 (3)	C51—C52—C53—C54	−1.0 (14)
O8—U—O5—N3	−179.0 (3)	O22—C21—C22—Cl4	3.8 (10)
O9—U—O5—N3	−104 (3)	N21—C21—C22—Cl4	−176.9 (6)
N4—U—O5—N3	−173.9 (3)	O22—C21—C22—Cl6	−124.2 (7)
U—O8—N4—O10	−176.7 (6)	N21—C21—C22—Cl6	55.1 (8)
U—O8—N4—O9	2.8 (6)	O22—C21—C22—Cl5	122.7 (7)
O3—U—N4—O10	−174 (100)	N21—C21—C22—Cl5	−58.0 (8)
O4—U—N4—O10	6(9)	C52—C53—C54—C55	−2.1 (18)
O21—U—N4—O10	96 (9)	C36—C31—C32—C33	2.7 (12)
O11—U—N4—O10	−83 (9)	N12—C31—C32—C33	−176.4 (8)
O6—U—N4—O10	−82 (9)	C32—C31—C36—C35	−3.8 (14)
O8—U—N4—O10	96 (9)	N12—C31—C36—C35	175.3 (9)
O9—U—N4—O10	−87 (9)	O12—C11—C12—Cl1A	−127.1 (9)
O5—U—N4—O10	87 (9)	N11—C11—C12—Cl1A	49.9 (9)
N3—U—N4—O10	20 (11)	O12—C11—C12—Cl2A	1.9 (11)
O3—U—N4—O8	89.7 (4)	N11—C11—C12—Cl2A	178.9 (8)
O4—U—N4—O8	−90.7 (4)	O12—C11—C12—Cl3B	147.6 (8)
O21—U—N4—O8	−0.9 (4)	N11—C11—C12—Cl3B	−35.4 (9)
O11—U—N4—O8	−179.7 (4)	O12—C11—C12—Cl2B	22.8 (10)
O6—U—N4—O8	−178.3 (4)	N11—C11—C12—Cl2B	−160.2 (8)
O9—U—N4—O8	177.0 (6)	O12—C11—C12—Cl3A	121.4 (10)
O5—U—N4—O8	−9.4 (6)	N11—C11—C12—Cl3A	−61.6 (9)
N3—U—N4—O8	−76 (3)	O12—C11—C12—Cl1B	−92.2 (9)
O3—U—N4—O9	−87.2 (4)	N11—C11—C12—Cl1B	84.8 (8)
O4—U—N4—O9	92.3 (4)	C46—C41—C42—C43	−3.6 (15)
O21—U—N4—O9	−177.9 (4)	N13—C41—C42—C43	180.0 (9)
O11—U—N4—O9	3.4 (4)	C53—C54—C55—C56	1.7 (19)
O6—U—N4—O9	4.7 (6)	C51—C56—C55—C54	1.7 (16)
O8—U—N4—O9	−177.0 (6)	C42—C41—C46—C45	1.0 (16)
O5—U—N4—O9	173.6 (4)	N13—C41—C46—C45	177.3 (9)
N3—U—N4—O9	107 (3)	C31—C36—C35—C34	2(2)
O21—P2—N22—C23B	158.1 (17)	C41—C42—C43—C44	2.2 (18)
N23—P2—N22—C23B	29.9 (18)	C42—C43—C44—C45	2(2)
N21—P2—N22—C23B	−86.1 (17)	C43—C44—C45—C46	−4.5 (19)
O21—P2—N22—C61A	−45.1 (14)	C41—C46—C45—C44	3.0 (18)
N23—P2—N22—C61A	−173.3 (14)	C31—C32—C33—C34	0.2 (18)
N21—P2—N22—C61A	70.7 (14)	C32—C33—C34—C35	−2(2)
O21—P2—N22—C61B	−32.5 (13)	C36—C35—C34—C33	1(2)
N23—P2—N22—C61B	−160.7 (12)	C23B—N22—C61A—C62A	−110 (3)
N21—P2—N22—C61B	83.3 (13)	C61B—N22—C61A—C62A	−3(12)
O21—P2—N22—C23A	154.7 (12)	C23A—N22—C61A—C62A	−112 (3)
N23—P2—N22—C23A	26.5 (12)	P2—N22—C61A—C62A	89 (2)
N21—P2—N22—C23A	−89.5 (12)	C23B—N22—C61A—C66A	66 (3)
O21—P2—N23—C51	105.4 (6)	C61B—N22—C61A—C66A	173 (16)
N22—P2—N23—C51	−131.5 (5)	C23A—N22—C61A—C66A	64 (3)
N21—P2—N23—C51	−10.3 (6)	P2—N22—C61A—C66A	−96 (3)
O21—P2—N23—C24	−65.5 (8)	C66A—C61A—C62A—C63A	0(2)
N22—P2—N23—C24	57.6 (8)	N22—C61A—C62A—C63A	176 (3)
N21—P2—N23—C24	178.7 (7)	C61A—C62A—C63A—C64A	2(3)
O11—P1—N13—C41	−130.9 (6)	C62A—C63A—C64A—C65A	−6(5)
N12—P1—N13—C41	105.1 (6)	C63A—C64A—C65A—C66A	8(6)
N11—P1—N13—C41	−14.7 (7)	C62A—C61A—C66A—C65A	3(4)
O11—P1—N13—C29	48.1 (7)	N22—C61A—C66A—C65A	−173 (3)
N12—P1—N13—C29	−75.9 (7)	C64A—C65A—C66A—C61A	−6(5)
N11—P1—N13—C29	164.2 (7)	C63B—C62B—C61B—C66B	0(2)
O10—N4—O9—U	176.7 (5)	C63B—C62B—C61B—N22	177 (2)
O8—N4—O9—U	−2.8 (6)	C23B—N22—C61B—C62B	−95 (2)
O3—U—O9—N4	93.5 (4)	C61A—N22—C61B—C62B	−172 (16)
O4—U—O9—N4	−86.6 (4)	C23A—N22—C61B—C62B	−94 (2)
O21—U—O9—N4	2.3 (4)	P2—N22—C61B—C62B	94 (2)
O11—U—O9—N4	−176.3 (4)	C23B—N22—C61B—C66B	82 (3)
O6—U—O9—N4	−177.4 (3)	C61A—N22—C61B—C66B	5(13)
O8—U—O9—N4	1.7 (3)	C23A—N22—C61B—C66B	83 (3)
O5—U—O9—N4	−75 (3)	P2—N22—C61B—C66B	−90 (2)
N3—U—O9—N4	−173.5 (4)	C61B—C62B—C63B—C64B	5(2)
U—O5—N3—O7	177.9 (6)	C62B—C63B—C64B—C65B	−15 (4)
U—O5—N3—O6	−0.3 (6)	C63B—C64B—C65B—C66B	20 (5)
U—O6—N3—O7	−177.9 (6)	C62B—C61B—C66B—C65B	5(4)
U—O6—N3—O5	0.3 (6)	N22—C61B—C66B—C65B	−171 (3)
O3—U—N3—O7	143 (12)	C64B—C65B—C66B—C61B	−15 (5)

### Hydrogen-bond geometry (Å, °)

**Table:**

*D*—H···*A*	*D*—H	H···*A*	*D*···*A*	*D*—H···*A*
N11—H11A···O6	0.86	2.06	2.811 (6)	146
N21—H21A···O8	0.86	2.11	2.861 (6)	146
